# 389. Epidemiology of Multiple Recurrent *Clostridioides difficile* in the Atlanta Metropolitan Area between 2016 and 2019

**DOI:** 10.1093/ofid/ofac492.467

**Published:** 2022-12-15

**Authors:** Scott Fridkin, Nirja Mehta, Colleen Kraft, Dana Goodenough, Stepy Thomas

**Affiliations:** Emory University School of Medicine, Atlanta, GA; Emory University, Atlanta, Georgia; Emory University, Atlanta, Georgia; Emory University School of Medicine, Atlanta, GA; Emory University, Atlanta, Georgia

## Abstract

**Background:**

Patients with multiple recurrences of *Clostridioides difficile* infection (CDI) have longer hospital stays and lower quality of life. Recent changes in therapies and strains of CDI make understanding recurrent CDI in the general population critical as these patients may benefit from microbiota restoring therapies rather than antibiotics alone.

**Methods:**

Georgia’s Emerging Infections Program (supported by CDC) conducts CDI surveillance in 8 counties around metropolitan Atlanta, GA (population ∼4 million). CDI is defined as any *C. difficile*-positive specimen with no positive test in the prior 2 weeks. We evaluated CDI between Jan 2015 through Dec 2019 and captured recurrent CDI (CDI test date 2-52 weeks following previous CDI) for 2016-2019 which were categorized as single episode only, recurrent (2 episodes ≤ 1 year) or multiple recurrent (>2 episodes ≤ 1 year). Year was attributed to date of final episode. Census data was used to determine crude and age-specific incidence. Bivariate and multivariable logistic regression was used to estimate odds for multiple recurrent compared to single recurrent CDI with demographic, comorbidity and treatment related covariates.

**Results:**

Over 4 years 13,745 patients had at least one episode of CDI, 2,930 (20%) had ≥ 1 recurrence and 916 (30%) of these progressed to multiple recurrence. Between 2016 to 2019, incidence of single CDI decreased 25% from 93/100,000 to 69/100,000 (P< 0.01). Multiple recurrent CDI decreased 45% from 9/100,000 to 5/100,000) (P< 0.01); incidence in the 80+ age group was highest and where decreased incidence was most dramatic during the study period (Figures). Time between 1^st^ and 2^nd^ episode was longer among patients with single recurrent than multiple recurrent CDI (median 12 weeks vs. 9 weeks, P< 0.01).. Independent predictors of multiple recurrence were fewer days (< 90) between episodes (aOR: 1.87 P< 0.01) and chronic renal disease (aOR: 1.59 , P< 0.01).

**Figure 1:**
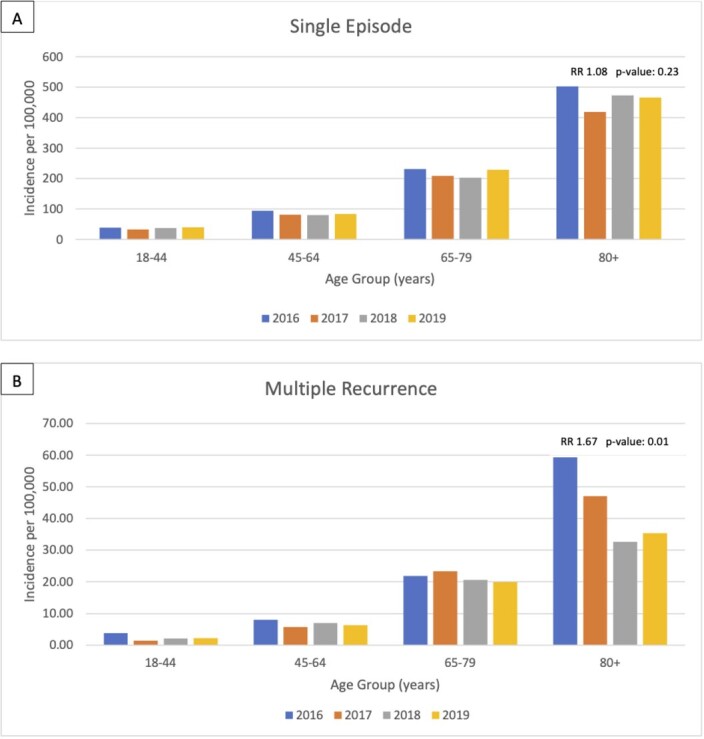
Annual Incidence of CDI by age group.

A) Incidence of CDI among patients with only a single episode of CDI within 365 days. B) Incidence of CDI among patients with three or more episodes (two or more recurrence) within 365 days. RR documented compares 2016 to 2018 incidence in the 80+ age group

**Conclusion:**

Time between 1st and 2nd CDI most strongly predicts likelihood of progression to multiple recurrences. Of all measured comorbid conditions, renal disease was most predictive. These findings may help to identify patients at high risk for progression for advanced interventions such as microbiota modifying therapies.

**Disclosures:**

**Scott Fridkin, MD**, Pfizer: Grant/Research Support **Colleen Kraft, MD MS**, Rebiotix Inc: Advisor/Consultant|SERES: Advisor/Consultant.

